# Two-step mechanism of Bruton’s tyrosine kinase membrane recruitment and activation

**DOI:** 10.1073/pnas.2528109123

**Published:** 2026-06-29

**Authors:** Rachel A. McAllister, Amy L. Stiegler, Keerthana Chari, Meera Chari, Moitrayee Bhattacharyya, Kallol Gupta

**Affiliations:** ^a^ Nanobiology Institute, Yale University, West Haven, CT 06516; ^b^ Department of Cell Biology, Yale University School of Medicine, New Haven, CT 06510; ^c^ Department of Pharmacology, Yale University School of Medicine, New Haven, CT 06510; ^d^ Department of Molecular Biophysics and Biochemistry, Yale University, New Haven, CT 06520

**Keywords:** native mass spectrometry, protein–lipid interaction, kinase regulation, B cell signaling

## Abstract

Peripheral membrane proteins (PMPs) are critical mediators of signaling cascades initiated at the cell surface. Their functions depend on their innate ability to interact dynamically with membranes in response to changing cellular conditions. This membrane recruitment may occur via high-affinity interactions with specific lipids/proteins or via transient, low-affinity interactions with the membrane. These weak and dynamic interactions, which are critical regulators of PMP function, are challenging to capture. Taking Bruton’s tyrosine kinase (BTK), a nonreceptor tyrosine kinase essential for B cell activation, we demonstrate a native mass spectrometry platform to understand lipid-mediated recruitment of PMPs by directly studying it from lipid bilayers customized to target membranes. Our data demonstrate that BTK recognizes phosphatidylserine (PS) independently of phosphatidylinositol (3, 4, 5) phosphate (PIP_3_) binding. We show that PS-bound BTK retains PIP_3_ binding via high-affinity sites, while exhibiting PIP_3_-independent basal membrane recruitment. Biochemical assays show that this PS-mediated recruitment sensitizes BTK to PIP_3_-mediated activation at near-physiological PIP_3_ concentrations. Thus, we propose a two-step model for BTK membrane recruitment and activation. A low-affinity interaction with high-copy number PS enables plasma membrane recruitment of BTK and increases its membrane-bound concentration. Upon B cell activation, this prerecruited, membrane-bound BTK population localizes to PIP_3_-rich domains via electrostatic gliding along the membrane, driven by low-affinity PS and high-affinity PIP_3_ binding. This indicates a cooperative mechanism in which PS can amplify B cell signaling by increasing membrane-bound BTK levels. Our work demonstrates a general model of regulation of PH domain–containing proteins by weak protein–lipid interactions, which can be extended to other PMPs.

## Significance

Specific recruitment of peripheral membrane proteins to the organellar membrane surface is essential for cellular signaling and maintaining organellar health. Often, these recruitments are guided by low-affinity, transient interactions with specific lipids on the organellar membrane surface that template the membrane-associated molecular complexes. We present a native mass spectrometry–based experimental platform to determine the lipid-guided recruitment mechanism of peripheral membrane proteins to the organellar surface by directly studying it from a customizable membrane. Applying this, we demonstrate how synergistic weak-affinity interactions with phosphatidylserine and high-affinity interactions of PIP_3_ regulate the recruitment and kinase activity of plasma membrane-bound Bruton’s tyrosine kinase.

**Fig. 1.**
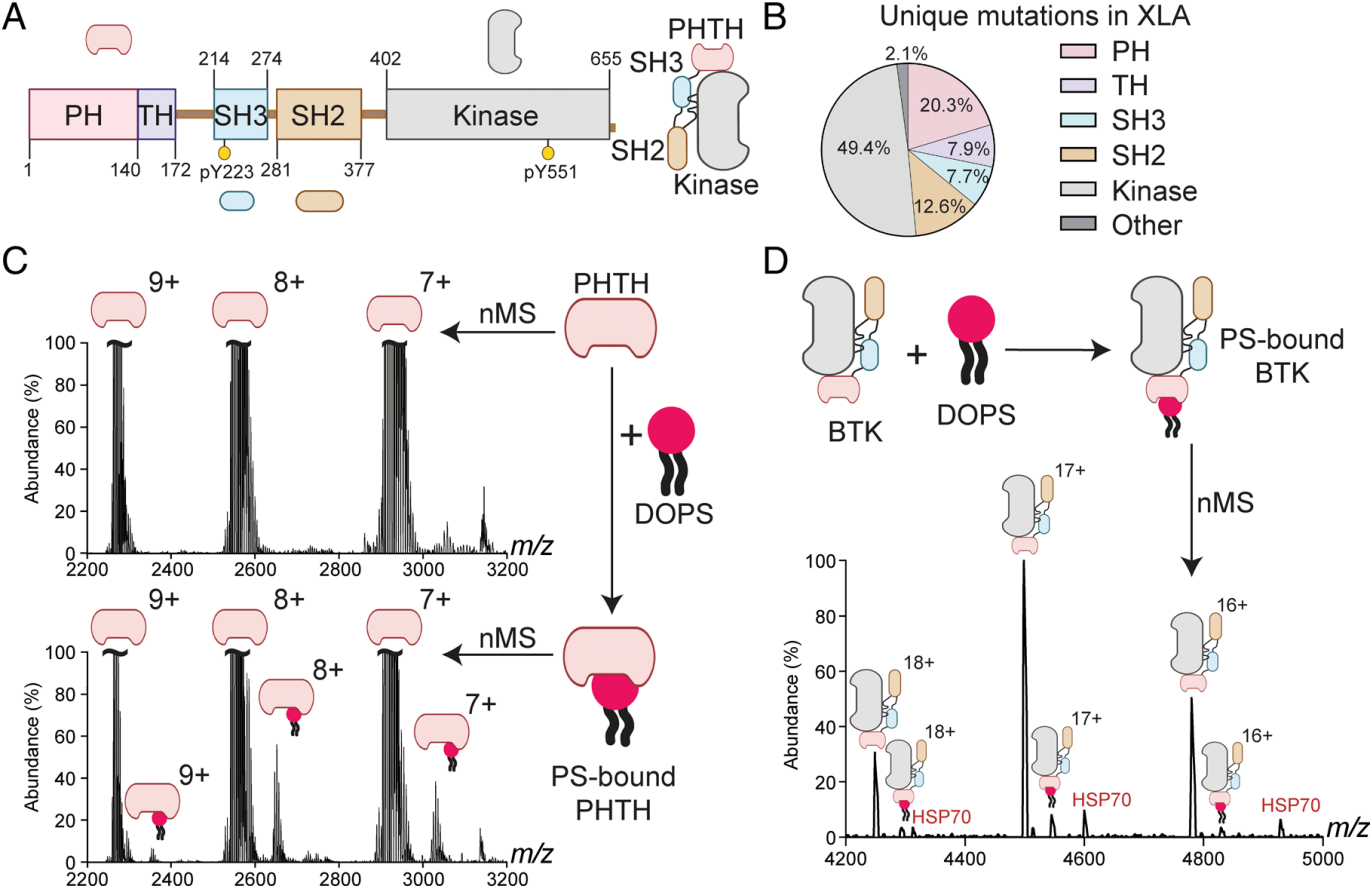
Structural features, XLA mutations, and PS binding of BTK: (*A*) Domain architecture of BTK showing the N-terminal PH and Tec Homology (TH) domain which contains a zinc finger motif, followed by an Src module composed of: an Src-homology 3 (SH3) domain, Src-homology 2 (SH2) domain, and a C-terminal kinase domain. Yellow circles denote major tyrosine phosphorylation sites Tyr223 and Tyr551. The structural model shows the autoinhibited state, in which the PHTH domain primarily folds against the kinase domain but can adopt a range of conformations (18, 20, 35). (*B*) Percentage distribution of unique X-linked agammaglobulinemia (XLA)-associated missense mutations mapped to each domain within BTK via *BTKbase* web-repository (32). (*C*) Native mass spectra of the isolated PHTH domain (*Top*) and in the presence of 18:1-18:1 phosphatidylserine (DOPS) (*Bottom*). Charge states are labeled and PS-bound peaks are marked. Observance of PS-bound peaks to all charge state peaks confirms the binding of PS to BTK. Spectra are normalized to 10% of major species in each spectra and repeated in triplicate. (*D*) Native mass spectra of the full-length BTK in the presence of DOPS. Minor purification impurities due to the presence of Heat Shock Protein 70 (HSP70) are annotated with a red HSP70. Like the isolated PHTH domain, the full-length BTK also binds to PS. The spectra were repeated in triplicate.

Cellular signaling pathways that initiate from organellar membrane receptors often rely on downstream peripheral membrane proteins to propagate the signals into cellular actions. This includes the Mitogen-Activated Protein Kinase (MAPK) pathway that regulates cell growth, the B cell receptor pathway that regulates immune response, and mitochondrial fission-fusion dynamics that regulate mitochondrial health (1–3). A significant subset of peripheral membrane proteins conditionally associates with membranes via specific recognition of signaling lipids such as phosphatidylinositol (3, 4, 5) phosphate (PIP_3_) (4–7). Among the most well-studied peripheral membrane protein domains is the Pleckstrin homology (PH) domain, the 11th-most-common domain in the human proteome (8). A key PH domain–containing peripheral membrane protein that plays an essential role in B cell receptor (BCR) signaling is Bruton’s tyrosine kinase (BTK) (9, 10). BTK is a Tec family kinase whose PH domain has an adjoining unique zinc finger motif required for structural stability (Fig. 1*A*) (11, 12). Upon antigen stimulation, the BCR undergoes clustering, leading to SYK/LYN-mediated phosphorylation of the immunoreceptor tyrosine-based activation motifs (ITAMs) on the associated immunoglobulins CD79a and CD79b (13, 14). This process initiates the downstream activation of phosphatidylinositol (3) kinase (PI3K), which phosphorylates membrane lipid phosphatidylinositol (4, 5)-bisphosphate (PIP_2_) to form phosphatidylinositol (3, 4, 5)-trisphosphate (PIP_3_) at the plasma membrane (15, 16). Previous studies have shown that PIP_3_ binding activates BTK, leading to Tyr223 autophosphorylation (12, 17). In the cytoplasm, BTK exists as an autoinhibited monomer with the SH2 and SH3 domains compact against the distal face of the kinase (Fig. 1*A*) (18, 19). On the membrane surface, PIP_3_ binding is suggested to directly compete with one autoinhibitory conformation of the PHTH domain in the full-length kinase and stabilize dimeric interfaces (20). This ability to relieve autoinhibition and stabilize BTK interactions is critical for BTK autophosphorylation at Tyr 551 in the kinase activation loop, thereby increasing kinase activity and subsequent trans-autophosphorylation at Tyr 223 in the SH3 domain (18, 21). Additionally, SYK or Src-family kinases, which are activated in parallel downstream of the BCR, can also phosphorylate Tyr551 in the kinase domain activation loop of BTK, further increasing its catalytic activity (13, 22). The SH2 domain of BTK can also recognize phosphotyrosines in peripheral membrane adaptor proteins such as B cell linker protein (BLNK) generated downstream of the BCR to help to bridge BTK with its downstream partners (23–25). Activated BTK phosphorylates and activates phospholipase-Cγ2 (PLCγ2), which cleaves PIP_2_ to produce diacylglycerol (DAG) and soluble secondary messenger inositol triphosphate (IP_3_) (26, 27). Additionally, mutations in the SH2 domain that prevent recognition of BLNK phosphotyrosine motifs have been found in X-linked agammaglobulinemia (XLA), a severe immunodeficiency syndrome (24, 25). Activated BTK phosphorylates and activates phospholipase-Cγ2 (PLCγ2), which cleaves PIP_2_ to produce diacylglycerol (DAG) and soluble secondary messenger inositol triphosphate (IP_3_) (26, 27). IP_3_ binds to the IP_3_ receptor in the endoplasmic reticulum (ER), releasing calcium and activating the nuclear factor of activated T cells (NFAT) and nuclear factor-kappa B (NF-κB) pathways, leading to antigen internalization and B cell activation (28–30). Due to BTK’s central role in adaptive immunity, mutations within its canonical PIP_3_-binding pocket have been shown to cause XLA (Fig. 1*B*) (31, 32). A key step in the BCR activation is the plasma membrane recruitment and activation of BTK (12, 21, 33, 34). While these SH2 domain-mediated protein–protein interactions discussed above (23–25) are well established, from the perspective of lipid-mediated recruitment to the plasma membrane, the current mechanistic understanding is that PIP_3_ alone is responsible for both the lipid-mediated recruitment of BTK to the plasma membrane and its activation in the membrane.

The central hypothesis of this study is that BTK can simultaneously interact with other abundant plasma membrane inner-leaflet lipids, in addition to PIP_3_. We propose that these likely weaker interactions promote PIP_3_-independent recruitment of BTK to the plasma membrane in an inactive state, increasing its local concentration and enabling BTK to scan along the quasi-two-dimensional inner leaflet of the plasma membrane for PIP_3_. Upon PIP_3_ generation, this membrane-prelocalized pool enables a stronger activation response, thereby amplifying PIP_3_-mediated BTK activation and subsequent signaling through orthogonal weak-affinity lipid interactions. Indeed, while PH domains were initially thought to primarily recognize PIPs, in recent years, studies have shown a broad range of functions, including work on the isolated PH domains of GRP1, ASAP1, and PDK1, which show that background electrostatic lipids induce higher membrane residence (36–39). These studies all suggest that a broader mechanism of coincidence detection of negatively charged phospholipids on the target membrane may play a significant role in recruiting peripheral membrane proteins for activation. Establishing this dual-mode lipid interaction requires experimental approaches that provide a dynamic detection range and molecular resolution to simultaneously detect both tightly and weakly bound lipids and unambiguously determine their identities and stoichiometry. Furthermore, it demands technical abilities to perform these analyses on formed target protein lipid complexes directly from a membrane environment that can be customized to a target physiological membrane. We have recently developed a native mass spectrometry (nMS) approach that enables the study of integral membrane proteins from customizable proteoliposomes (40, 41). This enables a quantitative understanding of the hierarchical organization of membrane proteins and their lipid-binding specificity directly from lipid bilayers mimicking different organellar membrane contexts. In this work, we further optimize and extend this platform to investigate how specific lipids regulate the recruitment of peripheral membrane proteins to organellar membranes, using BTK as the target protein. A key advantage of our nMS is its unmatched molecular resolution for detecting and identifying lipids specifically bound to target proteins—either individually or together—along with their binding stoichiometries, directly from complex lipid bilayers (40, 41). The general sensitivity of nMS enables further detection of even a minor fraction of protein bound to a lipid, allowing for application to a wide and dynamic range of protein lipid interactions (42–46). Using this lipid vesicle nMS platform, we found that BTK can be recruited to the membrane surface via a weak affinity interaction with phosphatidylserine (PS), independent of PIP_3_. We further established that this low-affinity interaction with PS, a highly abundant lipid on the inner leaflet of the plasma membrane, can occur independently of the PIP_3_ binding site or the recently proposed peripheral site. Using in vitro kinase assays, we further show that this PIP_3_ -independent PS-mediated recruitment leads to an increase in the degree of BTK autophosphorylation in the presence of a physiologically relevant amount of PIP_3_. This builds a two-step recruitment and activation mechanism for BTK, in which PIP_3_ and PS act synergistically. Beyond BTK, the work also presents a methodological platform for understanding the membrane recruitment of peripheral membrane proteins with precise molecular detail.

## Results

Our first goal was to determine if BTK can bind to other plasma membrane lipids beyond PIP_3_. To address this, we first used only the membrane-binding PHTH domain of BTK and studied independent lipid binding in solution using nMS. By incubating the PHTH domain with different lipids independently and subjecting it to nMS, we found that the PHTH domain binds PS (Fig. 1*C*), a negatively charged phospholipid highly abundant on the plasma membrane. PS is primarily localized to the inner leaflet, accounting for ~1/5th of inner leaflet lipids (47–51). In contrast, no binding was detected for phosphatidylethanolamine (PE), another prominent plasma membrane lipid enriched on the inner leaflet, indicating the specificity of the protein for the anionic serine head group over the more zwitterionic PE head group (*SI Appendix*, Fig. S1 *A*–*D*) (47, 51). In the full-length autoinhibited conformation of the protein, the PH domain is sandwiched against the N-lobe of the kinase, yet explores a range of conformations, leading to minor heterogeneity in the autoinhibited state (Fig. 1*A*) (18–20). Given the potential occlusion of PHTH domain surfaces, we wanted to confirm that full-length BTK can also recognize PS. As shown in Fig. 1*D*, full-length BTK also binds to PS, indicating that binding to PS is not abrogated by the PHTH domain conformation in the full-length protein. Finally, we aimed to demonstrate that this PS–BTK interaction can also occur in the presence of a bilayer, within the lipid-rich milieu that comprises the collective membrane environment. This is a critical consideration when studying protein–lipid interactions. While a target protein may bind a specific lipid in isolation, such interactions may not persist in the context of a physiologically relevant lipid bilayer (52). In these environments, other abundant lipids could potentially compete for binding, disrupt, or modulate the interaction (40, 41). Therefore, assessing lipid binding within native or native-like lipid mixtures is essential for accurately capturing biologically meaningful interactions.

**Fig. 2.**
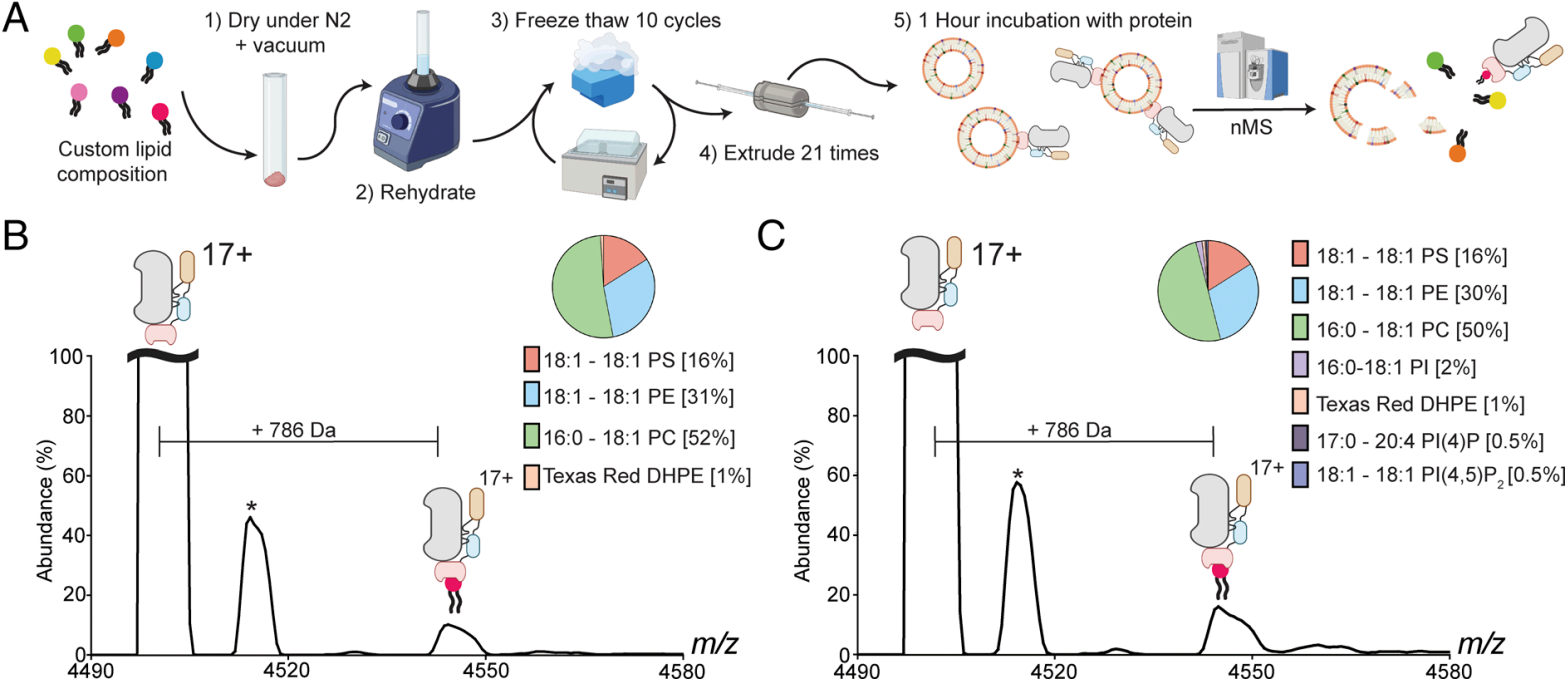
Lipid binding to BTK directly from the plasma membrane inner leaflet–like liposome. (*A*) Flow chart of peripheral membrane protein proteo-liposome preparation. Lipids in chloroform are dried under a nitrogen flow for 30 min, followed by a minimum of 2 h under vacuum to remove residual organic solvent. Aqueous nMS buffer is added to the dried lipid film for vortex rehydration, followed by 10 cycles of alternating liquid nitrogen and a 50 °C water bath, then extrusion through 100 nm pore membranes using an Avestin Liposofast basic. The homogeneous vesicles are then incubated with protein prior to nMS, during which the protein can be ejected from the vesicles, with the bound lipid still attached. (*B*) Mass Spectra of full-length BTK in the presence of PC/PE/PS vesicles. Lipid vesicle composition is shown on the *Right*. As annotated, direct and exclusive binding of PS to BTK was observed in the liposome. Spectra were normalized to 10% of the apo peak and repeated in triplicate. (*C*) Mass Spectra of full-length BTK in the presence of inner-leaflet plasma membrane-like vesicles. Lipid vesicle composition shown on the *Right*. Exclusive binding to PS to BTK was still observed, despite the presence of other inner-leaflet anionic lipids at physiological ratios. Spectra were normalized to 10% of the apo peak and repeated in triplicate. In both spectra, the * denotes a minor impurity of 241 Da addition that corresponds to a covalent phosphogluconylation of BTK. A noted and established artifact of overexpression of membrane-binding domains/proteins in *Escherichia coli* (7).

To address this, we incorporated our recently developed nMS method that enables the study of integral membrane protein–lipid interactions directly from customizable liposomes (40, 41). Here, we adapted this approach to peripheral membrane proteins, where gentle ionization and in-source activation disrupts the vesicles enough to resolve direct protein–lipid complexes (Fig. 2*A*). First, from liposomes containing phosphatidylcholine (PC), PE, and a minor amount PS—we observe a robust and exclusive PS binding peak (Fig. 2*B*). Next, we customized the lipid composition of the liposome to more closely resemble the lipid composition of the inner leaflet of the plasma membrane, the leaflet encountered by cytosolic peripheral membrane proteins. Typically, the inner leaflet is expected to be devoid of cholesterol and sphingomyelin, which primarily reside on the outer leaflet, and high PS (50, 51). As shown in Fig. 2*C*, even in the plasma membrane inner leaflet–like liposome containing seven different phospholipid species, PS binding is observed as the primary adduct. The ability to detect robust PS binding to BTK incubated with these custom lipid bilayers indicates the ability of PS in an inner leaflet–like bilayer to recruit BTK to the membrane surface.

Next, we asked whether PS binding to BTK can occur independently of PIP_3_ binding, since both PS and PIP_3_ are negatively charged lipids that may bind to similar positively charged residues on proteins. To test this, we employed dual mutations at the canonical PIP_3_ binding site formed by Lys 12, Arg 28, Asp 24, and Tyr 39 and performed nMS of this R28C/N24D mutant BTK, which has been shown to abolish the PIP_3_ binding to BTK (18, 31, 33). The mutation at R28C is one of the first prolific XLA mutant sites, with Arg 28 contacting the 3-phosphate, helping to bury the lipid head group within the loop cavities (11, 31). Crystal structures with IP_4_ show that all four residues (Lys 12, Arg 28, Asp 24, and Tyr 39) coordinate the phosphate groups (18). Hence, an additional mutation of Asn 24 to Asp, on top of R28C, helps reduce the positive charge by adding a negatively charged group within the PIP3 pocket itself (18). nMS-based lipid binding studies on this mutant reveal that it remains capable of binding to PS (*SI Appendix*, Fig. S2 *A*–*C*). We next tested a recently discovered secondary binding site for inositol hexakisphosphate (IP_6_), coordinated through Arg 52, Lys 36, Lys 49, and Tyr 40 (18). nMS of K49S/R52S mutant PHTH also shows no significant drop in the PS binding ability over the wild type (WT) protein (*SI Appendix*, Fig. S3 *A*–*C*). Together, these data indicate that BTK can bind to PS independently of intact PIP_3_-binding sites. Nevertheless, it may still be possible that these mutations do not completely abrogate PS binding to the PIP_3_-binding pocket. To further establish the independent dual-binding capabilities, we performed a series of dual-binding experiments using both full-length BTK and isolated PTHTH domains, as well as IP_4_, PIP_3_, and PS.

**Fig. 3.**
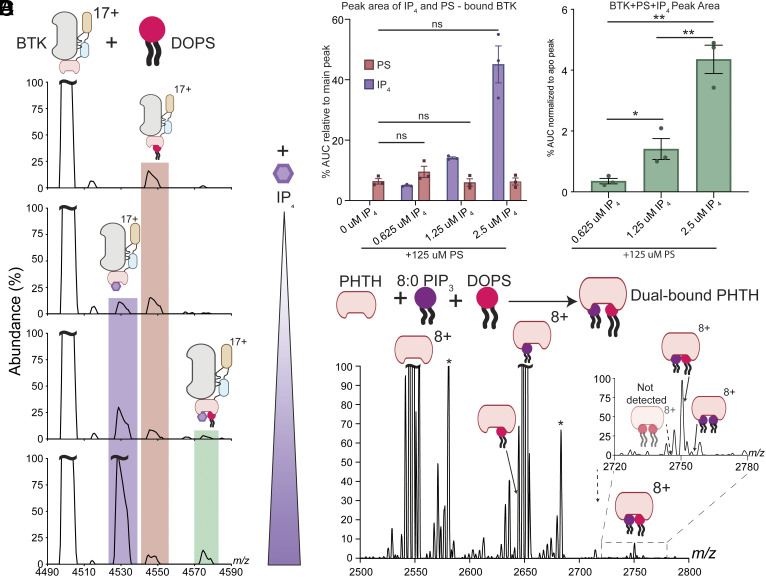
Ability of BTK to bind both PS and PIP_3_/IP_4_ simultaneously. (*A*) Expansion of charge state 17 of nMS of full-length BTK after incubation with DOPS. *Top* to *Bottom*: Increasing amounts of inositol tetrakisphosphate (IP_4_) are added. BTK is kept at 2.5 μM, PS at 125 μM, and IP_4_ concentration (*Top* to *Bottom*: 0 μM, 0.625 μM, 1.25 μM, and 2.5 μM). Peak abundance is normalized to 50% of the charge state 17 peak. As highlighted in a light fuchsia, all conditions produce a PS-bound peak. Incubation with IP_4_ yields a peak bound to IP_4_, highlighted in purple. Additionally, increasing the IP_4_ concentration yields a peak, highlighted in green, corresponding to the BTK + PS + IP_4_ species. (*B*) Plot of the peak area of the PS and IP_4_-bound peaks obtained from (*A*), highlighted in red and purple, respectively. The area under the curve (AUC) was calculated for each as a percentage of the apo charge-state peak (mean ± SEM, n = 3). Statistics calculated using an unpaired *t* test on GraphPad Prism. Statistical significance defined as *P* > 0.05, ns; *P* < 0.05 as *P < 0.01 as **P < 0.001 as ***. (*C*) Plot of the peak area of the peak at m/z = 4,574 corresponding to dual IP_4_ and PS-bound BTK (highlighted in light green) (mean ± SEM, n = 3). Statistics calculated using an unpaired *t* test on GraphPad Prism. (*D*) Spectra of the WT PHTH domain (4 uM) upon incubation with both PIP_3_ (2 uM) and PS (200 uM) reveal a unique peak at m/z 2,750, corresponding to the mass of PHTH bound to PIP_3_ and PS simultaneously. On the *Right*, the peak corresponding to the mass of PHTH + PIP_3_ + PIP_3_ is also annotated. No peak corresponding to PHTH + PS + PS (represented in faded Structure) was observed. The theoretical m/z value, where the hypothetical dual PS-bound peak should have been, is indicated with a dotted arrow. Spectra were normalized to 10% (100% of the primary peak in the *Inset*), and the experiments were performed in triplicate.

First, to unambiguously establish that such dual binding can occur directly from a membrane environment, we performed nMS of BTK in the presence of PS-containing liposomes, where we added IP_4_. IP_4_ is the functionally active headgroup of PIP_3_ that has been shown to adopt the same binding pose as PIP_3_ at the canonical site (*SI Appendix*, Fig. S4) (53). At a steady concentration of BTK and PS, we increased the IP_4_ concentration, and the resultant solution was analyzed by nMS. The apo and lipid/IP_4_ - bound BTK peaks were quantified via the area under the curve for the predominant charge state. As shown in Fig. 3*A* and quantified in Fig. 3 *B* and *C*, the addition of IP_4_ results in clear IP_4_-bound BTK and, critically, no significant loss of PS binding. More importantly, as the concentration of IP_4_ in solution increases, a distinct peak at m/z 4,574 arises, corresponding to the mass of BTK bound simultaneously to IP_4_ and PS. This indicates that PS-bound BTK can simultaneously bind to the PIP_3_ head group. Though structure and mutational analyses indicate that IP_4_ has a strong affinity for a primary PIP_3_ binding site (10, 12, 18, 54), we cannot completely rule out that IP_4_ recognizes additional sites within the protein. To rule this out we used the full-length intact PIP_3_ and incubated the PHTH domain with both PS and PI (3,4,5) P_3_. nMS of this mixture identified a characteristic peak corresponding to the simultaneous PIP_3_ + PS-bound state. Fig. 3*D* shows the 8+ charge states, where the peak at m/z 2,750 corresponds to the PIP_3_ + PS-bound peak. As also highlighted, this peak can be clearly distinguished from the dual PS or dual PIP_3_-bound species. This peak was absent in controls or when incubated with PS or PIP_3_ (*SI Appendix*, Fig. S5). Together, these data unambiguously establish that BTK can simultaneously bind PS while engaging PIP_3_ through the canonical binding site.

**Fig. 4.**
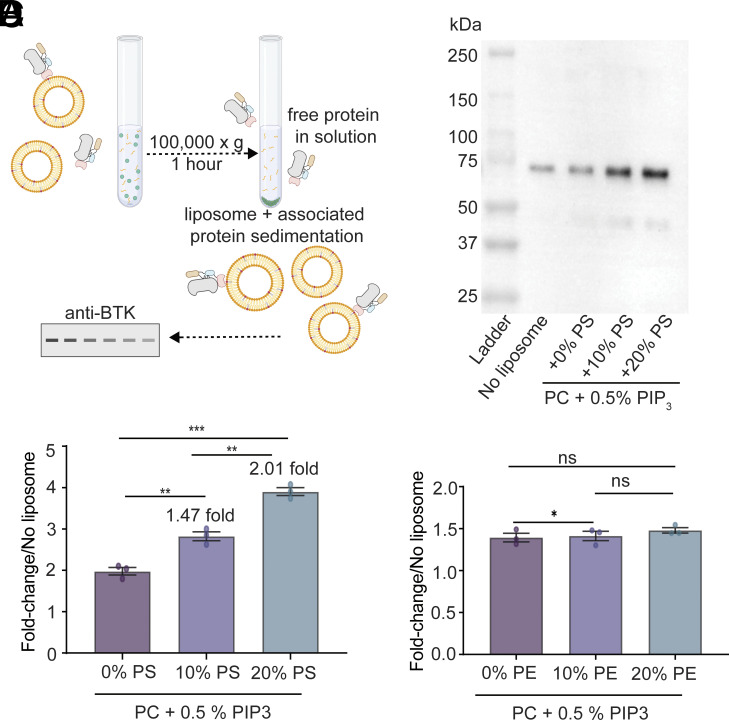
Membrane association of BTK as a function of PS concentration at low PIP_3_. (*A*) Cartoon schematic depicting the liposome sedimentation assay. Briefly, full-length BTK and liposomes are incubated for 30 min, followed by ultracentrifugation at 100,000 × g for 1 h. Liposomes, both with and without associated protein, are pelleted. The supernatant is removed, the pellet is collected, and the sample is run on an anti-BTK western blot to quantify protein. (*B*) Western blot showing the BTK concentration in pellets from sedimentation in the absence of liposomes (no liposome) and in the presence of liposomes comprised primarily of PC, 0.5% Texas Red DHPE, with 0.5% PIP_3,_ with PS values ranging from 0 to 20%. The concentration of PC only was adjusted to accommodate PS increases. The BTK band intensities are then quantified. (*C*) Plot showing quantification of BTK amounts in pellets, normalized by total BTK input, for each liposome condition with increasing PS%. The amounts are expressed as fold change relative to the no-liposome negative control (mean ± SEM, n = 3), which was set to 1. The average fold change in normalized pelleted BTK amounts in 10 and 20% PS liposomes relative to 0% PS is also noted on top of the bars. (*D*) Quantification of the BTK band found within the pellets of each liposome with increasing PE%, represented as fold-change increase relative to the no liposome negative control (mean ± SEM, n = 3), which was set to 1. The average fold change of normalized pelleted BTK amounts in 10 and 20% PE liposomes relative to 0% PE is also noted on top of the bars. Statistics calculated using an unpaired *t* test on GraphPad Prism. Statistical significance defined as *P* > 0.05, ns; *P* < 0.05 as *P < 0.01 as **P < 0.001 as ***.

We next sought to complement this observation by implementing a solution phase recruitment study of BTK to PS-containing bilayers using liposome sedimentation assays (Fig. 4*A*)(55, 56). Protein was incubated with liposomes containing PC and 0.5% PIP_3_, while the PS concentration varied from 0 to 20%. Protein without a liposome was used as a control and was also subjected to the sedimentation assay. For each condition, the amount of protein pelleted was estimated using western blot analysis. This amount was further normalized to the total input intensity to account for variability in sample amount (*Materials and Methods*, *SI Appendix*, Fig. S6). For each liposome condition, we used the normalized amount of pelleted protein to calculate the amount of membrane-bound BTK, expressed as a fold change relative to the no-liposome control (Fig. 4 *B* and *C*). Separately, the increase in BTK amount in 10 and 20% PS-containing liposomes over the 0% PS liposomes is marked on top of the respective bar charts. As shown in Fig. 4 *B* and *C*, the 0% PS liposome shows a similar amount of BTK pelleted as in the no-liposome control. Nevertheless, the association of BTK with liposomes increased with increasing PS percentage. 10 and 20% Ps containing liposomes show ~2.8 and ~3.9-fold increase in BTK sedimented over no liposome controls. As highlighted on top of the bar chart, this corresponds to a twofold increase in the amount of membrane-bound BTK when going from 0% PS to 20% PS (Fig. 4*C*). This unambiguously demonstrates that PS can play a significant role in the membrane association of BTK, and at low PIP_3_ concentrations, significantly enhances the local concentration of BTK near the membrane, with an expected increase in activity. As a control, we also subjected BTK to the same liposome assay, but this time increasing the mol % PE. As shown (Fig. 4*D*), increasing PE does not lead to an increase in BTK sedimentation, echoing the nMS-derived observation that BTK does not bind to PE. Further, we subjected R28C/N24D to the same experiment, and, as expected from the nMS-derived results, increasing PS% led to increased sedimentation of BTK (*SI Appendix*, Fig. S6). Together, these experiments establish that BTK can bind to PS at sites orthogonal to the PIP_3_ binding site while simultaneously binding to PIP_3_. This increases BTK membrane concentration in a PIP_3_ independent manner.

**Fig. 5.**
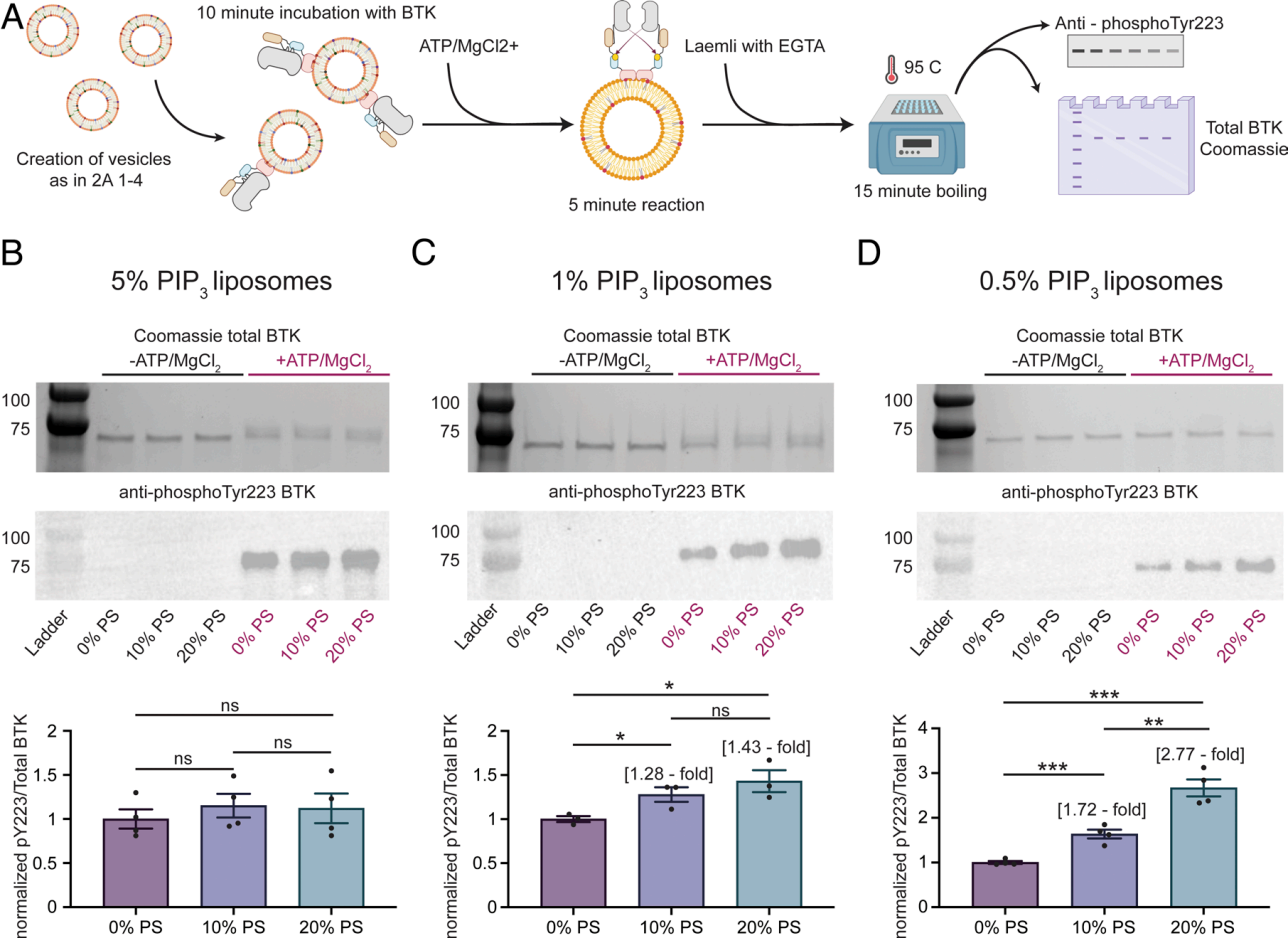
Influence of PS on the trans-*auto*phosphorylation of BTK. (A) Schematic depicting the kinase assay. Briefly, PC +PIP_3_ + PS liposomes are created following the protocol in Fig. 2*A*. PIP_3_ and PS concentrations are annotated in each figure, with the remainder mol% comprised of PC. After a 10-min preincubation with BTK, kinase reaction buffer is added and reaction proceeds for 5 min. The reaction was stopped by the addition of Laemmli w/ EGTA and boiling. For more details, see *Materials and Methods*. All conditions normalized to the mean of 0% PS for their respective PIP_3_ concentrations and representative westerns generated as a merge between colorimetric (ladder) and chemiluminescence. (*B, Top*) Representative Coomassie stain of 5% PIP_3_ liposomes to demonstrate total Btk concentration in both the negative control in the absence of ATP and MgCl_2_ as well as in the reaction conditions. (*Middle*) Representative anti-phosphoTyr223 BTK western. (*Bottom*) Plot of the quantified and normalized phosphoTyr223/Total BTK ratio (mean ± SEM, n = 4). No change in extent of phosphorylation observed despite changing PS concentrations. Statistical significance defined as *P* > 0.05, ns; *P* < 0.05 as * *P* < 0.01 as ** *P* < 0.001 as ***. (*C, Top*) Representative Coomassie stain of 1% PIP_3_ liposomes to demonstrate total Btk concentration in both the negative control in the absence of ATP and MgCl_2_, as well as in the reaction conditions. (*Middle*) Representative anti-phosphoTyr223 BTK western. (*Bottom*) Plot of the quantified and normalized phosphoTyr223/Total BTK ratio (mean ± SEM, n = 3). Increases in relative phosphorylation from 0 to 10% PS or 20% demonstrate an increase in BTK activation in the background presence of anionic PS. (*D, Top*) Representative Coomassie stain of 0.5% PIP_3_ liposomes to demonstrate total Btk concentration in both the negative control in the absence of ATP and MgCl_2_, as well as in the reaction conditions. (*Middle*) Representative anti-phosphoTyr223 BTK western. (*Bottom*) Plot of the quantified phosphoTyr223/Total BTK ratio (mean ± SEM, n = 4). At a 0.5% PIP_3_ concentration, a stark increase in autophosphorylation is observed as PS concentration increases, demonstrating a near-linear increase in BTK activity with increasing PS concentration.

Given that BTK activation critically involves trans-autophosphorylation, this PS-mediated increase in local BTK concentration on the plasma membrane is likely to facilitate autophosphorylation and enhance BTK activation, particularly under conditions of limited PIP_3_ availability. To assess the influence of PS-dependent membrane recruitment on BTK activation, we utilized an in vitro BTK autophosphorylation assay (18). We set up BTK phosphorylation assays in the presence of liposomes, modulating PS and PIP_3_ concentrations, with the remaining lipids as PC. For e.g., 5% PIP_3_ and 10% PS liposomes have 85% PC. BTK was incubated with custom liposomes before the introduction of ATP and MgCl_2_ to initiate the reaction. The reaction was stopped after 5 min by adding SDS buffer and EGTA, then boiling the sample (Fig. 5*A*). The extent of BTK autophosphorylation was measured by western blot using BTK phosphoTyr223 antibodies and normalized to total BTK concentration. Previous work has established that PIP_3_ is necessary and sufficient for activation of trans-autophosphorylation of BTK on the membrane (31, 57). While both Tyr223 and Tyr551 are autophosphorylation sites, Tyr223 is exclusively an autophosphorylation site and is commonly used as a readout of BTK activity in cells (17, 58). Although either can be used to measure in vitro phosphorylation, we used BTK Tyr223 because the corresponding phospho-antibody (pY223) was readily available to us.

Aligning with prior observation, PS and other phosphatidylinositol-containing liposomes (PIP or PIP_2_) failed to activate BTK by themselves on 0% PIP_3_ liposomes (*SI Appendix*, Fig. S7). Anionic lipids alone are incapable of increasing autophosphorylation relative to BTK in solution alone without any PIP_3_ present. This aligns well with previous mechanistic work demonstrating that stable membrane-bound PIP_3_ facilitates BTK autophosphorylation (12, 33). Next, BTK autophosphorylation assays conducted with liposomes containing high levels (5%) of PIP_3_ showed autophosphorylation, although no additional influence from increasing PS concentrations in the liposomes could be observed (Fig. 5*B*). However, under lower PIP_3_ concentration (1 to 0.5%), incremental increases in PS concentration led to a near-linear enhancement of BTK autophosphorylation (Fig. 5 *C* and *D*). Notably, the magnitude of this PS-mediated amplification of autophosphorylation inversely correlated with PIP_3_ abundance—reduction in PIP_3_ concentration heightened the impact of PS in the liposomes. Strikingly, at 0.5% PIP_3,_ we note a 2.77-fold increase in kinase autophosphorylation going from 0% PS to 20% PS (Fig. 5*D*). Based on our experimental data, we constructed a simple mathematical model to understand how PS-mediated membrane recruitment can increase the rate of BTK autophosphorylation (*Materials and Methods*). For this, we leveraged a previously established framework and further modified it to approximate how the effective concentration of BTK at the liposome surface would change in the presence of PS at low PIP_3_ concentrations (18). This simple, approximate model predicts a ~twofold increase in the autophosphorylation rate at 0.5% PIP_3_, going from 0 to 20% PS (*SI Appendix*, Fig. S8), closely matching the experimental data. Together, these results agree with our nMS-based observations and a two-step model: Low-affinity interactions with abundant PS enhance local BTK membrane concentrations, thereby increasing the ease and extent of BTK autophosphorylation at lower PIP_3_ concentrations.

## Discussion

Our findings reveal an interaction between BTK and PS, an abundant negatively charged lipid predominantly localized on the inner leaflet of the plasma membrane. Importantly, this interaction does not compete with canonical PIP_3_-binding. This allows BTK to simultaneously engage with PS and dynamically explore the membrane surface for high-affinity interactions with PIP_3_. We confirmed this through mutational analyses that selectively disrupted PIP_3_ binding without impairing PS interaction. Interestingly, the plasma membrane is composed of approximately 10% PS, with the majority localized to the inner leaflet, constituting about 15 to 20 mol% of inner leaflet lipids (47, 49). Given the very low physiological abundance of PIP_3_ (<1 mol%) and its short half-life, the observed low-affinity and high-copy-number PS interactions can substantially elevate local BTK concentration and facilitate rapid, dynamic membrane scanning by the PHTH domain for PIP_3_. For cytosolic peripheral membrane proteins like BTK, which interact exclusively with the inner leaflet, a lower-affinity interaction with an abundant lipid such as PS can facilitate dynamic exploration of the membrane. Beyond PIP_3_-mediated activation, these basal, nonactivating interactions can also enhance BTK’s search on the plasma membrane for other protein partners, such as BLNK, thereby facilitating BTK’s bridging to other components of the signaling nexus. The biological significance of this electrostatic interaction is highlighted by our kinase assays, which show that it enhances membrane-localized trans-autophosphorylation, thereby lowering the PIP3 threshold required to achieve robust BTK activation in response to upstream B cell receptor (BCR) signaling. Our liposome-based kinase assay showed a 2.77-fold increase in the extent of BTK autophosphorylation. Further mathematical modeling of the data corroborates this change. Conceivably, at higher PIP_3_ levels where the high-affinity PIP_3_ can fully saturate BTK, this effect of PS binding is expected to plateau. This is also supported by our kinase assay, which showed no effect of PS concentration at 5% PIP_3_-containing liposomes. At higher PIP_3_ concentrations, the excess PIP_3_, accounting for up to 1 in 20 lipids, begins to recruit much of BTK to the membrane, leaving less free solution-state BTK to be recruited to the surface by PS. This explains how differences in PIP_3_ concentration alter PS sensitivity to the kinase assay. Specifically, this PS-dependent interaction can position BTK favorably on the membrane surface, allowing its PH domain to efficiently search for and engage high-affinity activating ligands, such as PIP_3_. Importantly, this PS-mediated association with the basal membrane should also inherently elevate local BTK concentration at the plasma membrane, independent of PIP_3_ availability.

**Fig. 6.**
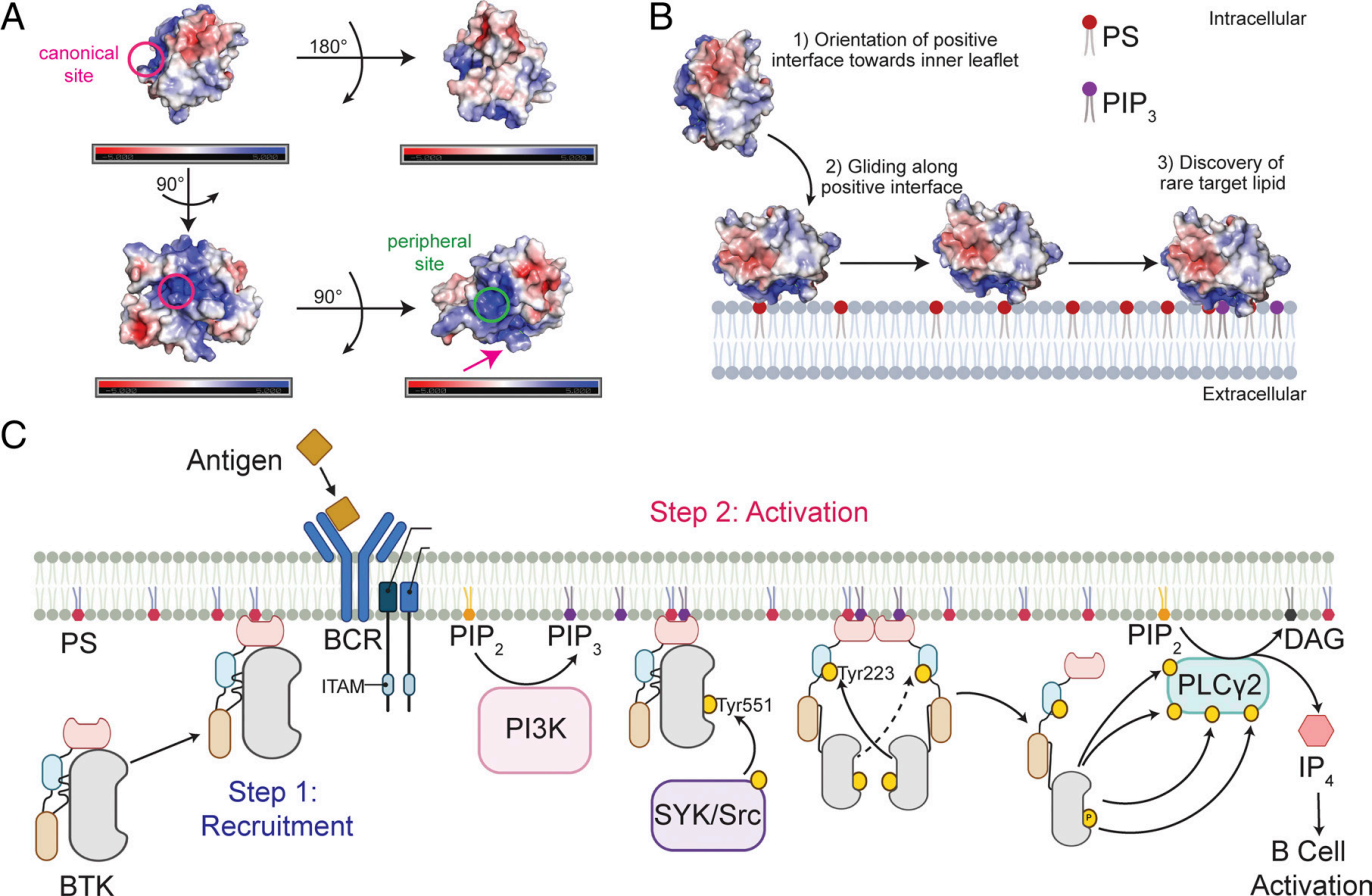
(*A*) (*Top Left*) Electrostatic map of the PHTH domain of BTK (PDB ID: 4Y94) (18). Electrostatics calculated on a single PHTH monomer using Pymol APBS electrostatics. Canonical site highlighted in fuschia circle (*Top Right*) Same plot orientated 180° around the x axis showing lack of electropositive character on this face. (*Bottom Left*) Plot from top left oriented 90° around the y axis, with the canonical PIP_3_ binding pocket highlighted in a fuschia circle. Large electrostatic positive character and pockets surround the canonical site. (*Bottom Right*) Plot from bottom left oriented 90° around the x axis highlighting another large electrostatic positive surface centered around the peripheral PIP_3_ binding site. Canonical site highlighted at the edge of the Structure with a fuschia arrow. Though the sites are two hotspots for binding PIP_3_, many electropositive lysines and arginines are exposed on proximal loops to these sites as seen in bottom left and bottom right though have not been directly implicated in coordinating PIP_3_ through the two defined sites. (*B*) Electrostatic map shown in (*A*) and model of membrane search mechanism, showing orientation of electropositive character surrounding the PIP_3_ binding site gliding along membrane, orienting PHTH domain towards the anionic lipids. (*C*) Schematic depicting the proposed two-step binding of BTK on the inner leaflet of the plasma membrane. First, due to a low-affinity interaction with the highly abundant PS, BTK is recruited to the surface of the plasma membrane inner leaflet to dynamically explore for a high affinity activating ligand. Upon BCR stimulation, this initiates a cascade that activates two critical upstream proteins: PI3K and Src-family kinases. PI3K converts lipid phosphatidyl inositol (4, 5) phosphate (PIP_2_) into phosphatidyl inositol (3–5) phosphate (PIP_3_). BTK is then activated by this high-affinity lipid and trans-autophosphorylates at Tyrosine 223 in the SH3 domain. Due to priming of BTK in the proximity of the membrane due to its recognition of PS, this activation can take place on a much faster scale. Src-family kinases, including LYN and FYN can phosphorylate Tyrosine 551 in the activation loop of BTK, increasing its catalytic activity. This fully active BTK can phosphorylate phospholipase C γ2, leading to cleavage of PIP_2_ to diacylglycerol and IP3, a secondary messenger leading to downstream BCR activation.

An unresolved aspect of this study is the precise delineation of PS binding sites on BTK. Despite extensive mutagenesis, we were unable to localize specific residues that disrupted PS binding. The PHTH domain contains a wide array of electrostatic residues in addition to charged amino acids that comprise the PIP_3_ binding pocket residues (*SI Appendix*, Fig. S9). These electrostatic residues are observed in the three major regions: the B1 to B2 loops, the B3 to B4 loop, and near the C-terminal helix. Among these, the charged residues after the C-terminus of a2 helix have been shown to recognize the N-lobe of the kinase, making these less likely to be involved in membrane association of the autoinhibited kinase. This finding raises the possibility that PS binding might not involve distinct, well-defined sites but rather general electrostatic interactions with the surface. Surface charge analysis further supports this hypothesis, demonstrating a large electropositive surface surrounding the canonical PIP_3_ site (Fig. 6*A* and *SI Appendix*, Fig S9). Such an extensive electropositive surface is likely to facilitate a generalized electrostatic mechanism, enabling PS-associated membrane-bound BTK to remain conformationally flexible, while gliding along the plasma membrane to present its canonical binding site optimally for high-affinity PIP_3_ interactions. While the current work does not pinpoint a specific binding site for PS, these structural features in BTK suggest possible modes of interaction. An interesting point to note is that in the cryoEM structure of the full-length BTK, the PHTH domain adopts a range of conformational states (20). The conformational flexibility of the PHTH domain, as evidenced by its heterogeneity in sampling positions around the Src kinase core, indicates that its orientation is largely mobile, supporting its ability to sample wide spatial regions. This also guides the PHTH domain to have the electrostatically positive surface facing the membrane, orienting the canonical PIP_3_ binding site toward the lipid bilayer (Fig. 6*B*). This orientation can facilitate faster search of the negatively charged inner leaflet and have the PHTH domain already orientated with the area around the canonical site toward the lipid head groups (Fig. 6*B*). Such an electrostatic gliding mechanism might allow BTK to rapidly scan the PS-rich inner leaflet of the plasma membrane for PIP_3_ (Fig. 6*C*). Indeed, previous computational and FRET-based studies on isolated PH domains of GRP1, a guanine nucleotide exchange factor, have proposed electrostatic hopping that can facilitate PIP_3_ binding (37). Notably, the nMS-based workflow provides the analytical precision required to unravel such nuanced multimodal interactions directly from lipid membranes, as exemplified by our successful demonstration of dual PIP_3_ and PS binding to BTK. The autophosphorylation assay further illustrates that these weak-affinity electrostatic interactions significantly reduce the threshold PIP_3_ concentration required to achieve physiologically relevant kinase activation upon BCR engagement. Given that the PH domain is one of the most widespread membrane-binding domains in the human proteome, found in over 250 distinct proteins, understanding how cells rapidly recruit PH-domain proteins using short-lived, low-abundant lipids such as PIP_3_ presents an exciting mechanistic question (59). Beyond the plasma membrane, negatively charged lipids differ substantially across organelles, such as phosphatidylglycerol and cardiolipin enriched in mitochondria, bis (monoacylglycero) phosphate in endolysosomes, or phosphatidic acid in nuclear membranes. Notably, these compartments also harbor PH domain–containing proteins. This raises the intriguing possibility that organelle-specific anionic lipids may analogously regulate the recruitment and activation of PH domain–containing proteins, akin to phosphatidylserine-dependent modulation of BTK at the plasma membrane. Our work establishes a broadly applicable and robust analytical framework for investigating multimodal peripheral membrane protein–lipid interactions. The nMS approach described herein, using a clinically relevant peripheral membrane protein, offers high molecular resolution, enabling the identification of protein–lipid interactions and providing a powerful tool for understanding the fundamental principles governing the selective recruitment of cytosolic effectors to various cellular membranes.

## Materials and Methods

### Protein Purifications.

Plasmids containing a His-SUMO tagged construct were transformed into *E. Coli* BL21 (DE3) cells for protein expression. The PHTH construct consisted of BTK residues 1 to 171. For full-length BTK, *E. Coli* cells also containing the expression constructs for tyrosine phosphatase (YopH) and GroEL/ES were generated and used. *Bos Taurus* BTK was used, as it retains >98% sequence homology and retains identical lipid binding sites and can be expressed in high quantities in *E. Coli*. Cells were grown in Terrific Broth (TB) with 70 µg/mL Kanamycin (with an additional 50 ug/mL Spectinomycin and 50 ug/mL Chloramphenicol for full-length BTK) to an optical density of 1.6 at 37 °C, at which point an equivalent amount of 4 °C TB with 70 µg/mL Kanamycin (with an additional 50 ug/mL Spectinomycin and 50 ug/mL Chloramphenicol for full-length BTK), 100 µM ZnCl_2_, and 1 mM IPTG was added to induce a 16-h expression at 18 °C. Cells were harvested at 4,000 × g for 30 min and cell pellets resuspended in Ni-NTA buffer A (25 mM Tris pH 8.5, 500 mM NaCl, 5% glycerol, and 20 mM imidazole) with protease inhibitor cocktail and DNAse. Cells were lysed via sonication (40% amplitude, 20 s on, 45 s off, 5 min total) and debris centrifuged out at 200,000 × g for 45 min at 4 °C. The supernatant was loaded onto a HisTrap 5 mL column using a peristaltic pump at 1 mL/min and washed with 10 column volume (CV) Ni-NTA buffer A. The protein was eluted via a gradient going from 0% Ni NTA Elution buffer (25 mM Tris pH 8.5, 500 mM NaCl, 5% glycerol, and 250 mM imidazole) to 100% over 5 CV increasing by 20% B for each CV. The eluted fractions containing protein were equilibrated into Desalting Buffer (25 mM Tris pH 8.0, 400 mM NaCl) on a HiPrep 26/10 desalting column to remove imidazole. The protein was incubated overnight with 500 µg His-tagged ULP1 protease at 4 °C to cleave the His-SUMO tag leaving the untagged protein and the cleaved His-SUMO and His-ULP1. Cleaved protein was then flowed over a HisTrap 5 mL column equilibrated in Ni-NTA buffer A to remove ULP1 and the His-SUMO product and collected in the flow-through. The flowthrough containing untagged protein was purified further using size exclusion chromatography (SEC) into SEC buffer (25 mM Tris-HCl, pH 8.0, 400 mM NaCl, 5% glycerol, and 1.5 mM TCEP) on a HiLoad 16/600 Superdex 200 pg column prior to concentration, aliquoting and flash-freezing.

### NativeMS.

For native mass spectrometry of the purified proteins, the proteins were buffer-exchanged into 500 mM ammonium acetate, 1 mM DTT using Zeba desalting columns (Thermo Fisher Scientific) at room temperature. Protein concentration was determined after exchange into the buffer. Protein ranges were kept between 1 and 5 µM, and electrospray was performed using in-house nano-emitter capillaries. These were generated by pulling borosilicate glass (O.D. –1.2 mm, I.D. –0.69 mm, 10 cm, Sutter Instruments) using a Flaming/Brown micropipette puller (Model P-100, Sutter Instruments) to a tip diameter within 4 to 5 µm. Platinum wire was inserted into the emitter and connected to the source to charge the sample. nMS was performed In a Q Exactive UHMR using a Nanospray Flex ion source (Thermo Fisher Scientific). Mass spectrometry parameters were optimized for each sample, for instance, spray voltage was in the range between 0.9 to 1.5 kV, the capillary temperature was 150 to 250 °C, the resolving power of the MS was in the range between 3,125 to 6,250 the in-source desolvation was between 50 V and 300 V. All nMS spectra were recorded with a 4.64 noise threshold cutoff—peaks presented and annotated are at least fivefold above the average noise level. All spectra were repeated in triplicate. Spectra are acquired from at least 100 independent scans, each of which is summed from at least three microscans. This ensures that every data point presented is averaged over at least 300 independent data scans, and only peaks more than fivefold higher than the standard noise level are carried forward for subsequent annotations. For incubation with lipids in detergent, lipids from Avanti Polar Lipids were dried under a nitrogen flow and under vacuum prior to resuspension by vortexing in 500 mM ammonium acetate + 1 mM DTT + 1.25× CMC OGNG. Protein and lipid were incubated for 1 h prior to being subjected to nMS analysis as described above. PHTH domain interactions with lipid were kept at 4 µM protein to 200 µM lipid. For the titration, FL BTK is kept at 2.5 μM, PS at 125 μM, and IP_4_ concentration at 0 μM, 0.625 μM, 1.25 μM, and 2.5 μM.

### Liposome Formation.

Lipids of appropriate concentrations were dried under nitrogen and vacuum for 1 h, then resuspended in 500 mM ammonium acetate supplemented with 1 mM DTT by vortexing for 1 h. Texas Red DHPE, as a minor component, was included to monitor vesicle stability during the extrusion process for nMS experiments. Lipid mixtures were then subjected to 10 freeze-thaw cycles and extruded using an Avestin liposofast extruder with 100 nm membranes to ensure homogeneity. BTK was prepared in 500 mM ammonium acetate and 1 mM DTT as described previously. BTK and respective liposomes were incubated at concentrations of 1.775 µM and 1.5 mM liposomes in 500 mM ammonium acetate and 1 mM DTT. For the detection of BTK in the presence of both liposomes and IP_4_, 0.85 µM IP_4_ was supplemented. IP_4_ was diluted to a working concentration in the same buffer prior to addition.

### Liposome Sedimentation Assay.

Lipids from Avanti were dried under nitrogen flow for 15 min, followed by an overnight vacuum to remove all leftover organic solvent. Dried lipids were rehydrated in liposome resuspension buffer (25 mM Tris-HCl, pH 7.4, 100 mM NaCl) to a concentration of 2 mM and subjected to 10 free-thaw cycles alternating between a 50 °C water bath and liquid nitrogen. These hydrated vesicles were extruded to 100 nm homogeneity using an Avestin LiposoFast. Full-length BTK at 2 µM and liposomes at 250 µM were incubated in BTK storage buffer B (25 mM Tris-HCl, pH 8.0, 400 mM NaCl, 5% glycerol, and 1.5 mM TCEP) for 30 min at room temperature. At this point, the sample was transferred to ultracentrifuge tubes and centrifuged at 100,000 × g for 1 h at 4 °C. An equal aliquot from the precentrifuge sample was set aside as the Total Input control (Loading control). The supernatant was collected, mixed with 5× Laemmli Buffer, and boiled. The pellet was resuspended in 1× Laemmli buffer, then boiled at 95 °C for 5 min. Samples were run on a SDS-PAGE gel (Bio-Rad 4 to 15% Mini-Protean gels) prior to transfer onto a PVDF membrane. Western membranes were blocked in 5% milk/TBST for 1 h at room temperature, then incubated with primary (1:20 k in 5% milk/TBST) and secondary (1:20 k in 5% milk/TBST). Westerns were imaged on a BioRad Chemidoc. For each condition, the intensity of the western blot readout of the BTK amounts in pellet was normalized to the total BTK input control western blot Intensity (loading control). Briefly, we first normalized the BTK amounts in pellets for each condition with the corresponding total BTK concentrations (i.e., total protein input) that were incubated with the liposomes before sedimentation. This gave us loading-control-normalized BTK amounts in the pellet across the four conditions reported (no liposome, 0%, 10%, and 20% PS). After this, we set the no-liposome-BTK pellet amount to 1 and calculated the respective fold changes in BTK pellet amounts for 0%, 10%, and 20% PS liposomes. We have also indicated the average fold change in BTK-pellet amounts for 10 and 20% PS (or PE) liposomes relative to 0% PS (or PE). All western blot data reported use this normalization procedure, ensuring that the total amount of protein input across different liposome conditions has remained the same in these experiments. Each experiment was performed in triplicate, with individual data points shown, and fold changes relative to the no-liposome control were plotted, with the mean fold change between conditions highlighted (mean ± SEM, n = 3).

### Kinase Activity.

Liposomes were dried under nitrogen and vacuumed, resuspended in liposome resuspension buffer (25 mM Tris-HCl pH 7.4, 100 mM NaCl) at 2 mM and subjected to ten free-thaw cycles using a 50 °C water bath and liquid nitrogen. These hydrated vesicles are then extruded to ensure 100 nm homogeneity using an Avestin LiposoFast. Full-length BTK at 4 µM and liposomes at 500 µM were prepared in kinase buffer (25 mM Tris pH 7.5, 150 mM NaCl, 5% glycerol) and subjected to a 10-min incubation at room temperature. This is taken as time point 0. At time point 0, an equal volume of kinase reaction buffer (25 mM Tris pH 7.5, 150 mM NaCl, 5% glycerol, 20 mM MgCl_2_, 2 mM ATP, and 2 mM sodium orthovanadate) or kinase negative control buffer (25 mM Tris pH 7.5, 150 mM NaCl, 5% glycerol, and 2 mM sodium orthovanadate) was added and the reaction proceeded for 5 min. All kinase and negative control reactions were stopped via the addition of an equivalent volume of 2× Laemmli sample buffer (Bio-Rad) with 100 mM EGTA and 5% β-mercaptoethanol and 15 min of 95 °C boiling.

*Auto*phosphorylation of BTK at phosphoTyr223 was measured using western blotting. Briefly, samples were run on an SDS-PAGE gel, before transfer to a PVDF membrane using a Tank transfer apparatus (BioRad). Membranes were blocked for 1 h rocking at room temperature in 4% Bovine Serum Albumin in Tris-buffered saline with tween (TBST). Overnight incubation in a 4 °C rocker with 1:2,000 (1:500 for 5% PIP_3_) phosphoTyr223 antibody in 1% BSA in TBST was followed by five 10-min TBST washes and secondary incubation with 1:5,000 (1:10,000) goat anti-mouse in 1% BSA in TBST and a sequential five 10-min TBST washes prior to imaging with SuperSignal West Femto or Pico PLUS Maximum Sensitivity Substrate (Thermo Fisher Scientific). For Coomassie staining, the gels were placed into fixation solution (40% methanol, 10% acetic acid), microwaved for 30 s and placed on a room temperature rocker for 30 min. Then, the gels were microwaved for 30 s in QC colloidal Coomassie stain (BioRad) and placed on a room-temperature rocker overnight before rinsing several times with water to destain. Both chemiluminescence and Coomassie blue staining were imaged on a BioRad Chemidoc. A prestained ladder was run to determine molecular weight and imaged in the westerns. Analysis and integration of the bands was performed on Fiji.

### Mathematical Modeling.

Mathematical model adopted from Wang et. al. (18). Previous estimates of effective BTK concentration (C) on the membrane surface were calculated as follows:

First, the active volume where BTK can operate within the lipid vesicle context, or the shell of the vesicle containing BTK is calculated as V = A × L (*SI Appendix*, Fig. S8*A*). In the model, A is the surface area of the vesicle, and L is the length of BTK, around 100 Å as in the BTK composite full-length structure. From this, C can be calculated as C = $\frac{N_{\mathrm{Btk}}}{VxN_{a}}$ where N_BTK_ is the number of BTK on a vesicle and N_a_ is Avogadro’s number. N_BTK_ is calculated from the molar fraction (f) of PIP_3_ and the total number of lipids in the vesicle (N_0_). N_0_ is given by A × A_0_, the surface area of a lipid head group, at roughly 0.6 nm^2^ (60).

C can then be calculated as

$$C=\frac{N}{VXN_{0}}=\frac{fX\frac{A}{A_{0}}}{AXLXN_{a}}=\frac{f}{A_{0}XLXN_{a}}.$$

From here, we can modify this equation for our kinase assays, to become

$$C=\frac{f_{1}+f_{2}\left(P\right)}{A_{0}XLXN_{a}},$$

where f_1_ represents molar fraction of PIP_3_, f_2_ represents molar fraction of PS and P represents the percentage of PS occupied by BTK. We then estimate P (the fraction of PS bound) based on our experimental data, and assuming the bulk of IP_4_ (PIP_3_ analog) is bound in the 1:1 stoichiometry as in the mathematical model. In our experimental titration with 0.625 µM IP_4_ we are using exactly 200-fold the concentration of PS as IP_4_ and achieving roughly twofold (1.916) the area of bound BTK. This approximates to around 1 in 100 PS is bound to BTK using the PIP_3_ analog for control. Our values become f_1_ = 0.005, 0.01, 0.025, and 0.05 according to PIP_3_ concentrations, f_2_ = 0, 0.1, and 0.2 accordingly *P* = 0.01 for 1 in 100 bound, A_0_ = 60 Å^2^, N_a_ = 6.03 × 10^23^ mol^−1^.

## Supplementary Material

Appendix 01 (PDF)

## Data Availability

All data are publicly available through the MASSIVE database under the Accession code: MSV000102128 (61).
