# Supplementary material for: Two-step mechanism of Bruton’s tyrosine kinase membrane recruitment and activation
Source: Proc Natl Acad Sci U S A. 2026 Jun 29;123(27):e2528109123. doi: 10.1073/pnas.2528109123 (PMC13342989; doi:10.1073/pnas.2528109123)
Supplement: Supplementary file 1 — Appendix 01 (PDF) [file pnas.2528109123.sapp.pdf]

PNAS

Supporting Information for

Two-Step Mechanism of Bruton's Tyrosine Kinase Membrane Recruitment and Activation

Rachel A. McAllister[1,2,3], Amy L. Stiegler[4], Keerthana Chari[1,2], Meera Chari[1,2], Moitrayee Bhattacharyya[3], Kallol Gupta[1,2]

*Co-corresponding authors: Kallol Gupta, Moitrayee Bhattacharyya

1) Nanobiology Institute, Yale University, West Haven, CT, USA, 06516
2) Department of Cell Biology, Yale University School of Medicine, New Haven, CT, USA, 06510
3) Department of Pharmacology, Yale University School of Medicine, New Haven, CT, USA, 06510
4) Department of Molecular Biophysics and Biochemistry, Yale University, New Haven, CT, USA, 06520

Email: kallol.gupta@yale.edu, moitrayee.bhattacharyya@yale.edu

This PDF file includes:

Figures S1 to S9

Figures

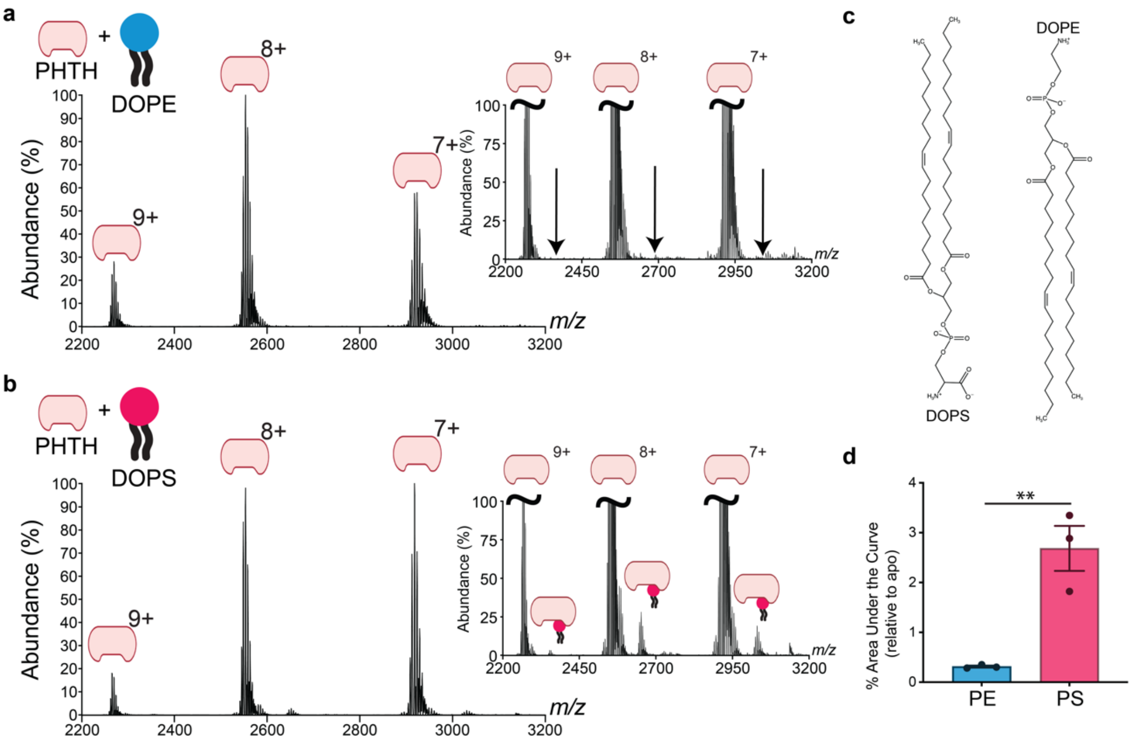

Fig. S1. PHTH domain recognition of PS. (a) Native mass spectra of the isolated PH-TH domain after incubation with 18:1-18:1 phosphatidylethanolamine (DOPE). No PE-bound peaks are identified. Arrows indicate predicted *m/z* for PE-bound peaks. Inset shows normalization to 10% of primary peak. **(b)** Native mass spectra of the isolated PHTH domain in the presence 18:1-18:1 phosphatidylserine (DOPS) under the same conditions reveals PS binding. Inset shows normalization to 10% of primary peak. **(c)** Schematics showing the structure of DOPS (left) and DOPE (right). The phospholipid backbones are identical, indicating any difference in behavior is specific to the head group. **(d)** Quantification of the lipid binding, shown as area under the curve relative to the apo 8+ charge state (mean ± sem, n=3). Statistics calculated using unpaired t-test on Graphpad Prism. Statistical significance defined as p>0.05, ns; p< 0.05 as *, p<0.01 as ** and p<0.001 as ***.

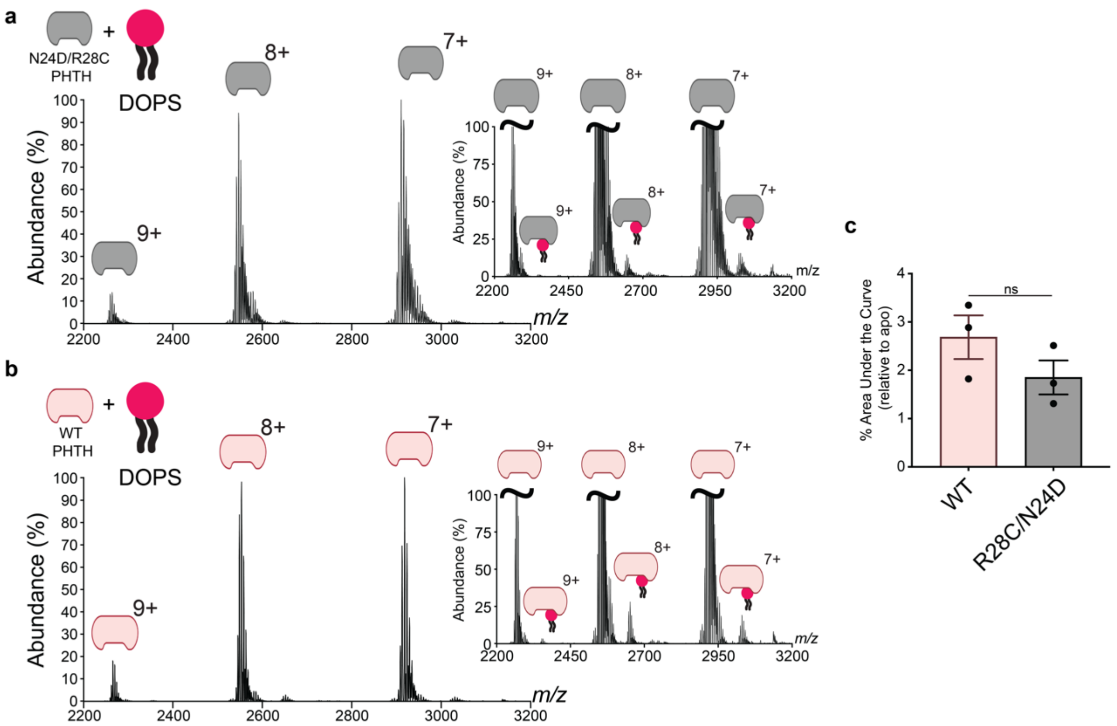

Fig. S2. Canonical site (R28C/N24D) mutant PHTH domain retains PS binding: (a) Native mass spectra of the isolated R28C/N24D mutant PHTH module in the presence of 18:1-18:1 phosphatidylserine (DOPS). Inset shows normalization to 10% of primary peak. **(b)** Native mass spectra of the isolated wild-type PHTH module in the presence of DOPS. Conditions are the same as in (a). Inset shows normalization to 10% of primary peak. **(c)** Area under the curve calculated relative to the primary charge state (8+) (mean ± sem, n=3). Statistics calculated using unpaired t-test on Graphpad Prism. Statistical significance defined as $p>0.05$, ns; $p< 0.05$ as *, $p<0.01$ as ** and $p<0.001$ as ***.

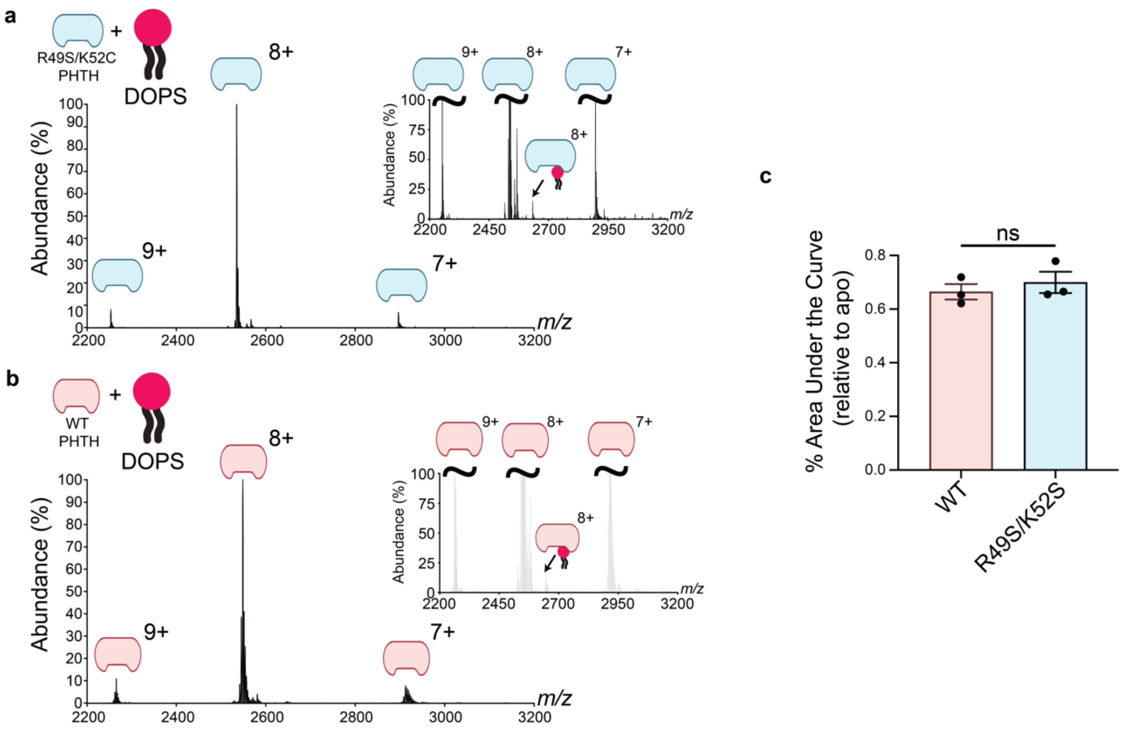

Fig. S3. Peripheral site mutant PHTH domain retains PS binding: **(a)** Native mass spectra of the isolated K49S/R52S mutant PHTH module in the presence of 18:1-18:1 phosphatidylserine (DOPS). Inset shows normalization to 5% of primary peak. **(b)** Native mass spectra of the isolated wild-type PHTH module in the presence of DOPS. Conditions are the same as in (a). Inset shows normalization to 5% of primary peak. **(c)** Area under the curve calculated relative to the primary charge state (8+). The area under the curve for each lipid-adducted species is calculated as a percentage of that same apo peak (mean ± sem, n=3). Statistics calculated using unpaired t-test on Graphpad Prism. Statistical significance defined as p>0.05, ns; p< 0.05 as *, p<0.01 as ** and p<0.001 as ***.

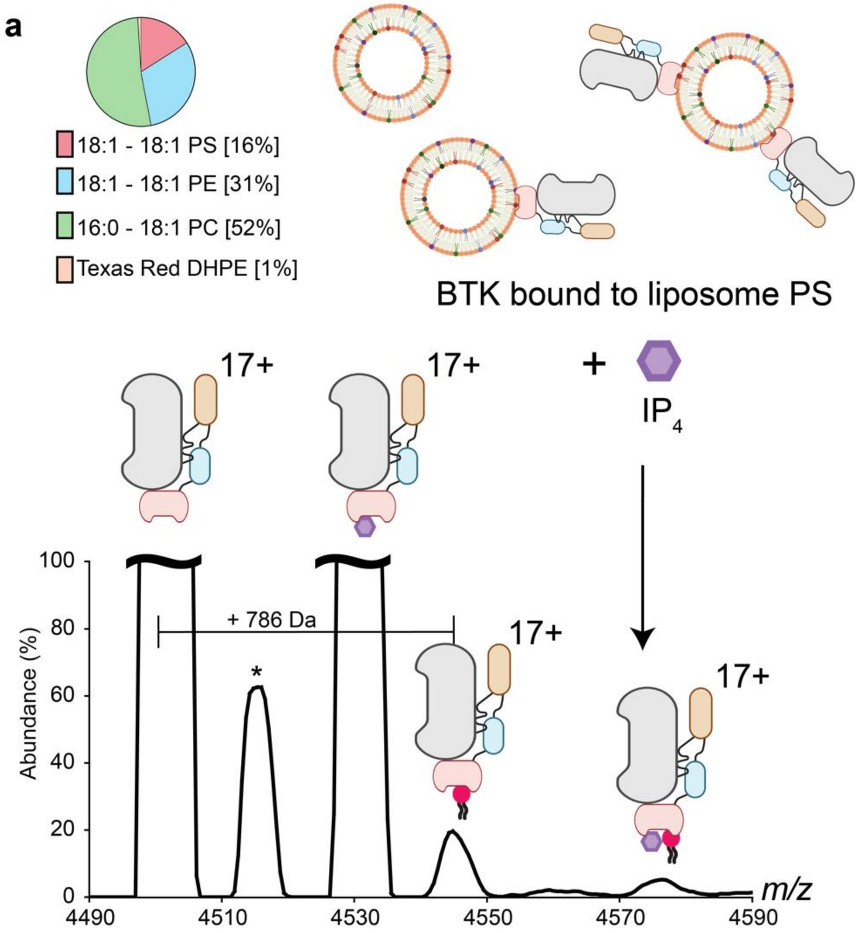

Fig. S4. BTK bound to membrane PS and IP_4: (a) nMS of BTK in the presence of liposomes containing PC/PE/PS and IP_4. Spectra highlights the binding of BTK to IP_4 and PS both individually and in unison.

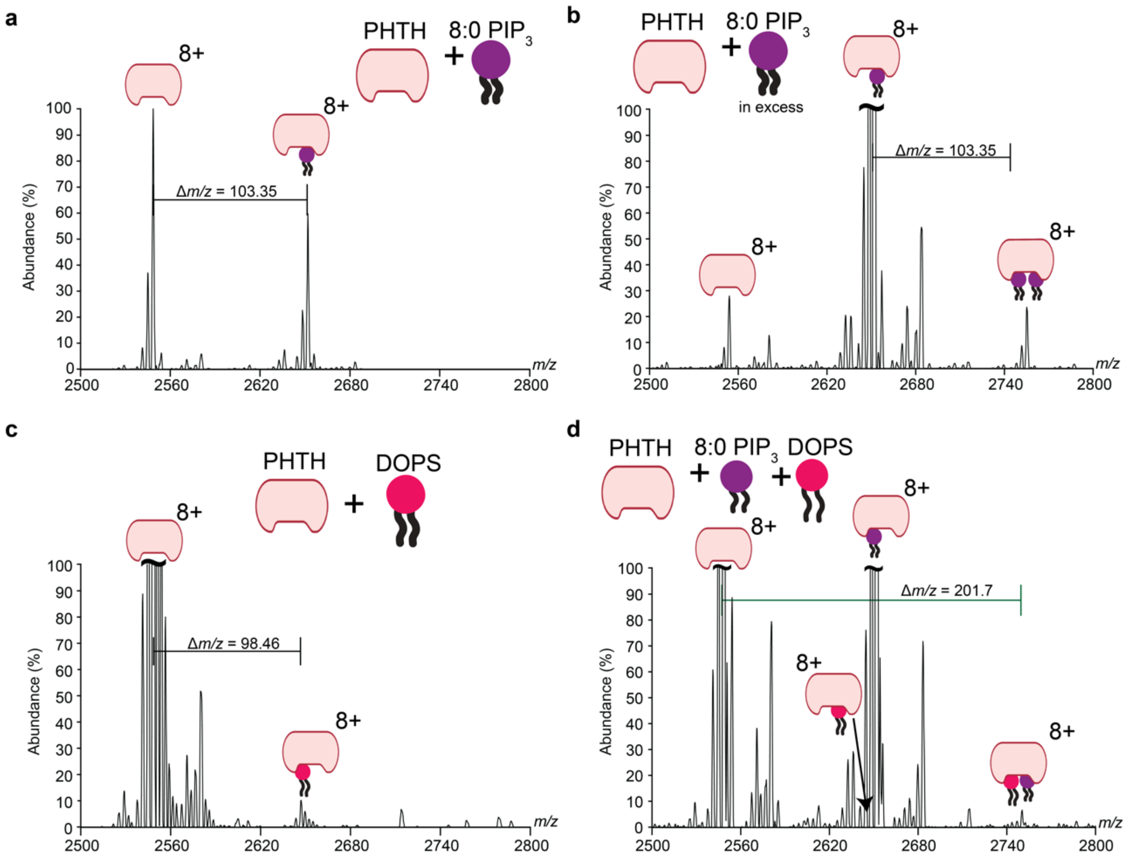

Fig. S5. Supplemental to dual binding of BTK PHTH to PS and PIP$_3$. (a) PHTH domain in the presence of 8:0 PIP$_3$ and no PS. The mass addition of 8:0 PIP$_3$ leads to a *m/z* difference of 103.35Th (2651.95 – 2548.6) between the apo and lipid-bound charge states. **(b)** PHTH domain in the presence of excess 8:0 PIP$_3$ and no PS, normalized to 10% of highest peak in the spectra. Binding of a second 8:0 PIP$_3$ species reveals a peak at 2755.35 *m/z*, at another addition of 103.4Th *m/z.* **(c)** PHTH domain in the presence of DOPS only normalized to 10% of the base peak in the spectra. The mass addition of DOPS leads to a *m/z* difference of 98.46Th (2647.16 – 2548.7) between the apo and lipid-bound charge states. **(d)** PHTH domain in the presence of 8:0 PIP$_3$ and PS, normalized to 10% of the base peak in the spectra. A peak exists at 2750.4 *m/z* at a 201.7Th *m/z* addition to the apo peak. The peak corresponding to two 8:0 PIP$_3$ bound simultaneously would be at 2755.35 *m/z* as shown in b. The predicted *m/z* for two DOPS simultaneously bound would be at 2745.62 *m/z*. The actual peak (2750.4 *m/z*) aligns with one of each lipid being bound.

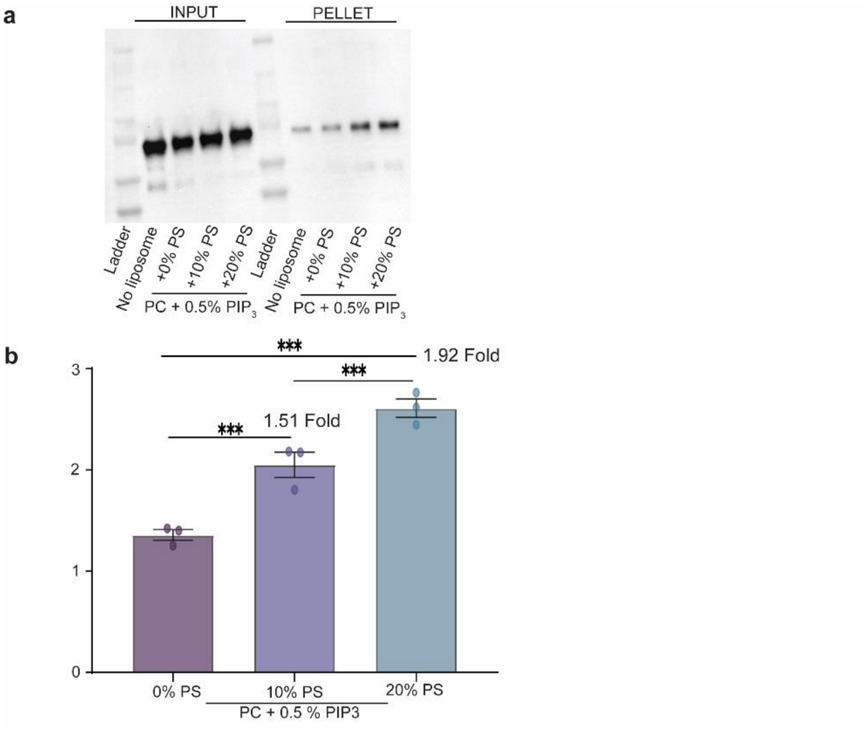

Fig. S6. Membrane association of WT and R28C/N24D BTK as a function of PS concentration at low PIP$_3$. (a) Western blot-based analysis of the liposome sedimentation assay of WT BTK, with the left-most panel showing the ladder, followed by four panels representing the input samples of no liposome, 0% PS liposome, 10% PS liposome, and 20 % PS liposome. This is followed by another ladder band, and the rest of the four bands represent pellets of the respective no liposome conditions with increasing PS %. **(c)** Plot showing quantification of BTK amounts in pellets, normalized by total BTK input, for each liposome condition with increasing PS %. The amounts are expressed as fold change relative to the no-liposome negative control (mean ± SEM, n=3), which was set to 1. The average fold change in normalized pelleted BTK amounts in 10% and 20% PS liposomes relative to 0% PS is also noted on top of the bars. Statistics calculated using unpaired t-test on Graphpad Prism. Statistical significance defined as $p>0.05$, ns; $p< 0.05$ as *, $p<0.01$ as ** and $p<0.001$ as ***.

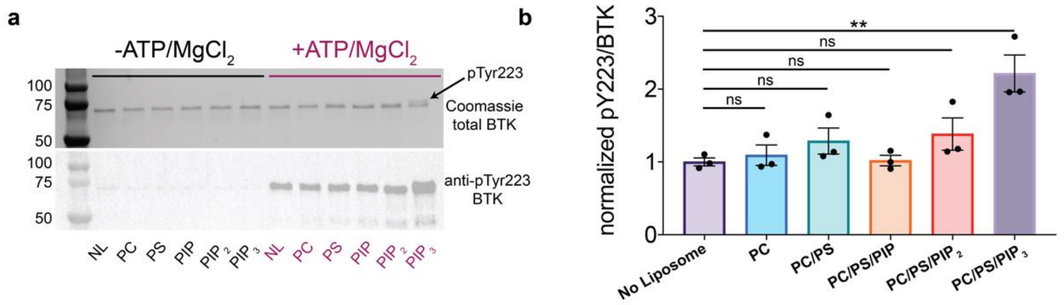

Fig. S7. Anionic Lipids alone do not activate BTK. (a) Kinase assay to demonstrate the effect of anionic lipids in the absence of PIP_3 on the kinase activity of BTK. Liposome lipid concentrations are NL – No liposome, PC – 100 mol %, PC/PS – 60/40 mol %, PC/PS/PIP – 87/10/3 mol %, PC/PS/PIP2 – 88/10/2 mol %, PC/PS/PIP3 – 88.5/10/1 mol %. **(top)** Representative Coomassie stain of each condition to demonstrate total Btk concentration in both the negative control in the absence of ATP and $MgCl_2$ as well as in the reaction conditions. The upper migrating band indicating the presence of phosphorylation in the 1% PIP_3 is highlighted with an arrow and annotated as pTyr223. **(bottom)** Representative anti-phosphoTyr223 BTK western. **(b)** Quantification of the BTK phosphoTyr223 relative to total BTK in each condition. All data shown as mean ± sem from n = 3 independent replicates. Results shown as unpaired t-test. Statistical significance defined as $p>0.05$, ns; $p< 0.05$ as *, $p<0.01$ as ** and $p<0.001$ as ***.

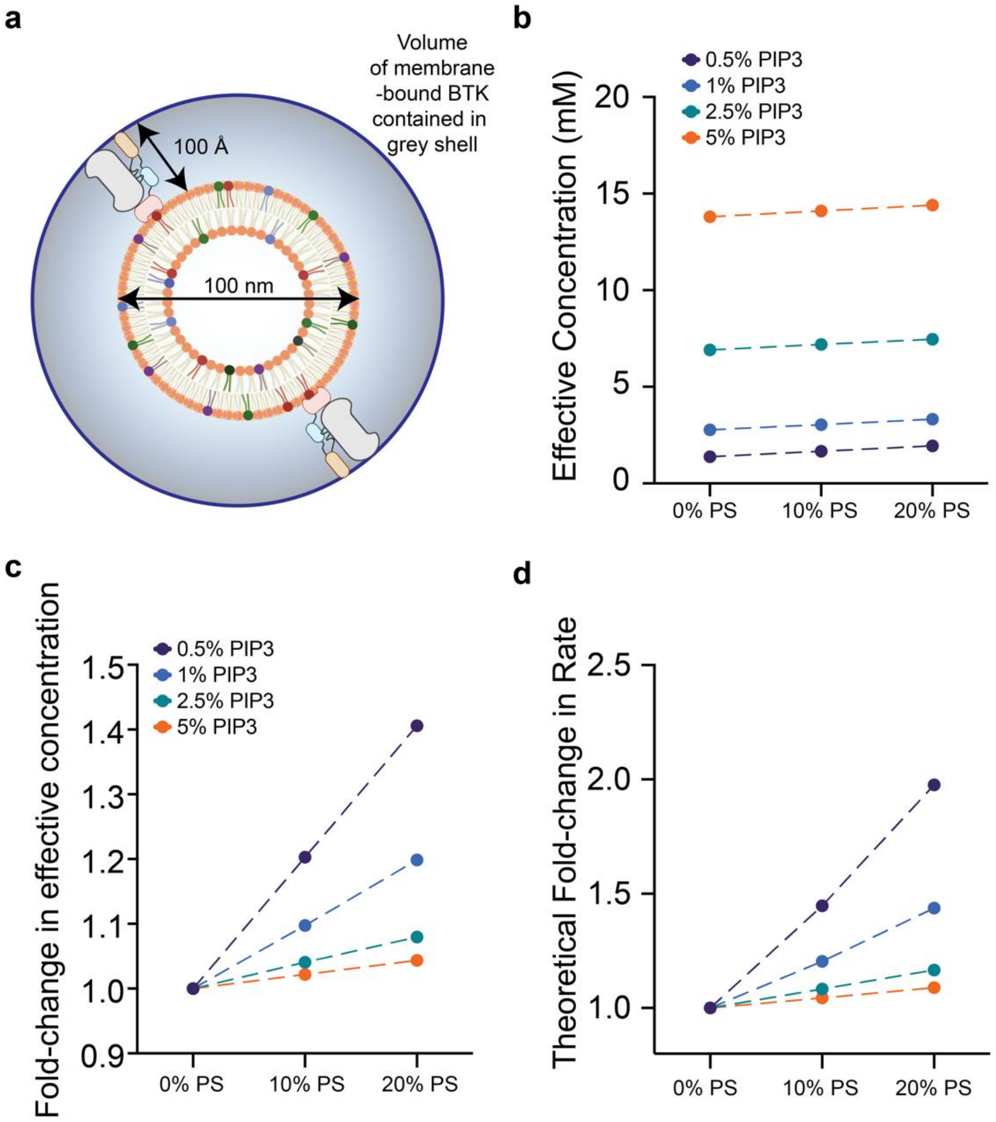

Fig. S8. Mathematical model for membrane-bound BTK concentration. (a) Model demonstrating how volume is calculated using the diameter of the lipid vesicle and the predicted length of full-length BTK. **(b)** Changes in the calculated effective concentration using the mathematical model for membrane-proximal BTK as a function of a set PIP_3 and changing PS concentration. As the PS concentration changes, the mM change is consistent. **(c)** Changes in the fold-change of effective concentration relative to 0% PS for each PIP_3 concentration. At lower PIP_3 concentrations, this leads to a more drastic change in the effective concentration. **(d)** Theoretical fold-change in rate calculated as the square of the fold-change in effective concentration, proportional to $[BTK]^2$. This provides an even more drastic increase as we consider the [BTK] as both the enzyme and substrate in a kinase autophosphorylation reaction.

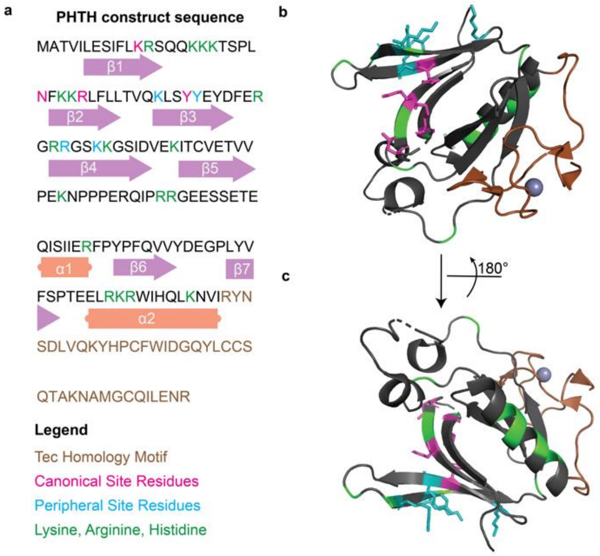

Fig. S9. The PHTH domain sequence and Structure. (a) The PHTH domain sequence is annotated for the Tec Homology Motif, binding-site residues, and charged residues. The C-terminus of the PHTH domain comprises the zinc-binding Tec Homology motif. Two sites, the canonical (higher-affinity) and peripheral (lower-affinity) sites for inositol phosphate recognition, have been structurally characterized, and residues are highlighted in pink and teal, respectively. Charged residues not implicated majorly for either binding pocket (lysine, arginine, histidine) are highlighted in green. (b) The Structure of the PHTH domain (PDB ID: 4Y94) in grey with annotations from the (a) sequence highlighted in identical colors. (c) Structure shown in (b) rotated 180 degrees around the x-axis.
